# Plasma levels of the pro-inflammatory protein S100A12 (EN-RAGE) are associated with muscle and fat mass in hemodialysis patients: a cross-sectional study

**DOI:** 10.1186/1475-2891-13-48

**Published:** 2014-05-27

**Authors:** Hirotaka Fukasawa, Sayaka Ishigaki, Naoko Kinoshita-Katahashi, Hideo Yasuda, Hiromichi Kumagai, Ryuichi Furuya

**Affiliations:** 1 Renal Division, Department of Internal Medicine, Iwata City Hospital, 512-3 Ohkubo, Iwata, Shizuoka, Japan; 2 First Department of Internal Medicine, Hamamatsu University School of Medicine, 1-20-1 Handayama, Hamamatsu, Shizuoka, Japan; 3 The Department of Clinical Nutrition, School of Food and Nutritional Sciences, University of Shizuoka, 52-1 Yada, Shizuoka, Shizuoka, Japan

**Keywords:** Hemodialysis, Malnutrition, Protein-energy wasting, S100A12, sRAGE

## Abstract

**Background:**

Malnutrition is highly prevalent and contributes to mortality in hemodialysis (HD) patients. Although the receptor for advanced glycation end products (RAGE) system also contributes to the morbidity and mortality of these patients, the role that the RAGE system plays in determining nutritional status is currently unknown.

**Methods:**

A cross-sectional study examining 79 HD patients was performed. The plasma concentrations of the soluble RAGE (sRAGE) and S100A12 (also known as EN-RAGE) were studied to evaluate their association with nutritional status, which was assessed by measuring the mid-thigh muscle mass and subcutaneous fat mass with computed tomography.

**Results:**

Plasma S100A12 concentrations were shown to be significantly and negatively correlated with muscle mass and with fat mass (*r* = −0.237, *P* < 0.05 and *r* = −0.261, *P* < 0.05, respectively). In contrast, sRAGE was not shown to significantly correlate with either of these factors. Multiple regression analyses demonstrated that S100A12 is a significant independent predictor of both muscle mass and fat mass (*P* < 0.01 and *P* < 0.05, respectively).

**Conclusions:**

Our findings suggest that plasma S100A12 levels could play an important role in determining muscle mass and fat mass in HD patients.

**Trial registration:**

Study number; UMIN000012341.

## Background

Protein-energy wasting (PEW), which is also referred to as malnutrition, is a condition caused by chronic kidney disease (CKD) and is characterized by decreased body stores of protein and energy sources [1]. PEW is present in a large proportion of advanced CKD patients, in whom it has been associated with atherosclerotic cardiovascular disease (CVD), and it has been shown to lead to increased morbidity and mortality [2,3]. Inadequate nutrition, inflammation, perturbations of appetite-controlling hormones, insulin resistance, enhanced proteolysis, and metabolic acidosis may contribute to the pathogenesis of PEW [4-6].

In the general population, overweight and obesity have been shown to be significant risk factors for cardiovascular and all-cause mortality [7]. In contrast, a higher body mass index (BMI) is associated with decreased mortality and a reduced risk of hospitalization in hemodialysis (HD) patients [8,9].

The receptor for advanced glycation end products (RAGE) has emerged as a central regulator of inflammatory processes [10]. This multiligand receptor is expressed on the cell surface, where it can bind to various types of ligands, including S100/calgranulins, high-mobility group box 1 (HMGB1) and advanced glycation end products (AGEs) [11]. The interactions between RAGE and its ligands activate pro-inflammatory genes, which can cause a wide range of inflammatory diseases, such as diabetic complications [11], rheumatoid arthritis [12], inflammatory bowel disease [13], and Alzheimer’s disease [14].

RAGE accumulates and exists in several forms in patients with decreased renal function [15,16]. Soluble RAGE (sRAGE) is a circulating form of RAGE that is likely generated by the shedding of RAGE bound to the cell surface. sRAGE has been hypothesized to act as a decoy receptor by competitively inhibiting the binding of RAGE ligands to RAGE, thus attenuating downstream inflammatory responses [17]. One protein that has been reported to be a ligand for RAGE is S100A12, which is also known as extracellular newly identified RAGE-binding protein (EN-RAGE). In contrast to sRAGE, S100A12 is a natural pro-inflammatory ligand for RAGE [18,19]. By releasing pro-inflammatory cytokines, such as interleukin (IL)-1β and tumor necrosis factor (TNF)-α, S100A12 activates inflammatory responses [18,20]. Moreover, it enhances the activation and migration of monocytes/macrophages. Consequently, S100A12 has been suggested to be an important contributor to the development of inflammatory processes, such as atherosclerosis [11,20].

Several studies have reported associations between the RAGE system and CKD. For example, Kim et al. [21] reported that sRAGE is negatively associated with systemic inflammation and carotid atherosclerosis in peritoneal dialysis (PD) patients. In addition, Nakashima et al. [22] and Shiotsu et al. [23] reported that S100A12 might be a strong predictor for CVD and mortality in HD patients. However, the potential relationship between the AGEs-RAGE system and nutritional status has not been reported in any field, including CKD.

In this study, we report cross-sectional data from a well-characterized cohort of patients undergoing maintenance HD. In addition, we assess the relationships between plasma RAGE levels and nutritional status by measuring the areas of mid-thigh muscle and subcutaneous fat in these patients.

## Materials and methods

### Subjects

Seventy-nine patients (53 men, 26 women) who had been undergoing HD at Iwata City Hospital (Shizuoka, Japan) were enrolled in this cross-sectional study. The causes of end-stage kidney disease were primary kidney diseases, such as chronic glomerulonephritis and nephrosclerosis in 65 patients (82%), polycystic kidney disease in six patients (8%), and overt diabetic nephropathy in 8 patients (10%). All patients had been subjected to regular HD for 4–5 hours three times per week at a blood flow rate of 180–240 ml/min. All patients used bicarbonate dialysate (Kindaly AF-2E^®^, Fuso, Osaka, Japan) at a dialysate flow rate of 500 ml/min. The study protocol was approved by the institutional ethics committee, and all patients provided informed consent before participating in the study.

### Anthropometric measurements

Body weight was measured before and after each dialysis session, and the post-dialysis body weight of each patient was used as his or her dry weight (DW). BMI (kg/m^2^) was calculated by dividing the DW (kg) by the squared height (m).

### Blood sampling and laboratory examinations

Blood samples were drawn at the beginning and end of the first dialysis session of the week, following a 2-day interval. Plasma samples were separated immediately and stored at -80ºC until analyzed. Serum electrolytes, urea nitrogen, creatinine (Cr), albumin, cholesterol, triglyceride, and C-reactive protein (CRP) levels were measured using standard laboratory techniques with an auto-analyzer. Plasma S100A12 and sRAGE levels were measured using enzyme-linked immunoassays (the CircuLex S100A12/EN-RAGE ELISA kit; CycLex, Nagano, Japan, and the Human RAGE Quantikine ELISA Kit; R&D Systems, Minneapolis, MN, USA, respectively). A single-pool urea kinetic model was used to calculate the protein catabolic rate and the delivered dialysis dose, [clearance of urea (*K*; mL/min) multiplied by the time on dialysis (*t*; min) divided by the volume of distribution for urea (*V*_urea_; mL)], as described by Depner and Daugirdas [24].

### Measurements of muscle and fat areas by computed tomography (CT)

Axial CT images of the thigh were obtained at the midpoint of a line extending from the superior border of the patella to the greater trochanter of the femur [25,26]. Each patient was examined in the supine position with his or her thigh muscle relaxed. The thickness of each slice was 10 mm. Radiographic images were digitally scanned for analysis with a personal computer. The adipose-tissue-free thigh muscle area (TMA) and thigh subcutaneous fat area (TSFA) were measured using NIH-IMAGE, a public domain planimetry program available from the National Institutes of Health (written by Wayne Rasband, The National Institutes of Health, Bethesda, MD, USA). To avoid the potentially confounding influence of body size, we also standardized TMA and TSFA by dividing by DW (TMA/DW ratio and TSFA/DW ratio, respectively) [26].

### Statistical analysis

Data were expressed as the mean ± standard deviation (SD) for continuous variables, with normal distributions or the median and interquartile range (25th to 75th percentiles) for data with skewed distributions. The threshold for statistical significance was set at *P* < 0.05. Comparisons between two groups were performed using the Mann–Whitney *U*-test, and comparisons between the three groups were made using the Kruskal-Wallis test. Spearman’s rank-order correlation analysis was used to evaluate the potential associations between TMA/DW, TSFA/DW, sRAGE, or S100A12 with the selected parameters. Multivariate regression analyses were used to assess the independent predictors of TMA/DW and TSFA/DW. All statistical analyses were performed using SPSS statistical software, version 19.0 (SPSS Inc., Chicago, IL, USA).

## Results

### Clinical profiles

Table 1 presents the characteristics of the study population. The median age was 67.0 years (the 25th to 75th percentile ranged from 60.0 to 73.5 years). The median dialysis vintage was 138.0 months (range, 43.5 to 268.0 months), and the mean BMI was 20.5 ± 2.9 kg/m^2^.

**Table 1:** Patient Characteristics

**Variables**	**Total (n = 79)**	**Men (n = 53)**	**Women (n = 26)**
Age, years	67.0 (60.0 to 73.5)	68.0 (60.0 to 74.0)	65.5 (59.8 to 71.5)
Dialysis vintage, months	138.0 (43.5 to 268.0)	134.0 (40.0 to 267.0)	157.5 (85.8 to 282.5)
Height, cm	160.2 ± 8.8	164.2 ± 6.3	152.1 ± 7.5^c^
Dry weight, kg	50.7 ± 9.8	56.5 ± 9.4	43.5 ± 7.4^c^
BMI, kg/m^2^	20.5 ± 2.9	20.9 ± 2.8	19.6 ± 3.0^a^
Total protein, g/dL	6.4 ± 0.4	6.4 ± 0.4	6.4 ± 0.4
Serum albumin, g/dL	3.7 ± 0.4	3.6 ± 0.4	3.7 ± 0.4
Total cholesterol, mg/dL	152.1 ± 34.3	145.1 ± 33.9	166.3 ± 31.1^a^
LDL choresterol, mg/dL	85.2 ± 21.1	82.5 ± 21.4	90.8 ± 19.6
Blood urea nitrogen, mg/dL	62.6 ± 12.7	64.8 ± 10.9	58.2 ± 15.1^a^
Serum creatinine, mg/dL	10.8 ± 2.7	11.2 ± 3.0	9.8 ± 1.7^b^
Calcium, mg/dL	9.4 ± 0.9	9.3 ± 0.9	9.7 ± 0.8
Phosphate, mg/dL	5.0 ± 1.3	5.1 ± 1.4	4.8 ± 1.2
Intact PTH, pg/mL	102.5 ± 107.4	90.5 ± 59.2	127.0 ± 166.6
β_2_-microglobulin, mg/L	26.7 ± 6.6	26.4 ± 6.6	27.3 ± 6.7
Kt/V_urea_	1.6 ± 0.3	1.5 ± 0.2	1.9 ± 0.3^c^
nPCR, g/kg/ideal body weight/day	0.97 ± 0.17	0.98 ± 0.15	0.94 ± 0.21
CRP, mg/dL	0.1 (0.0 to 0.3)	0.1 (0.1 to 0.4)	0.0 (0.0 to 0.1)
sRAGE, pg/mL	1550.4 (1005.3 to 2336.5)	1496.3 (950.0 to 2407.9)	1704.4 (1072.6 to 2240.6)
S100A12, ng/mL	32.3 (22.4 to 53.5)	33.4 (24.1 to 56.9)	25.3 (18.6 to 39.3)
TMA, cm^2^	75.7 (62.7 to 95.2)	88.2 (69.0 to 99.7)	60.9 (54.8 to 72.6)^c^
TMA standardized for DW	1.53 (1.41 to 1.75)	1.57 (1.44 to 1.79)	1.45 (1.24 to 1.63)^a^
TSFA, cm^2^	33.3 (23.2 to 47.2)	27.2 (19.6 to 40.8)	46.9 (31.1 to 59.4)^c^
TSFA standardized for DW	0.64 (0.47 to 0.87)	0.53 (0.42 to 0.67)	1.05 (0.75 to 1.39)^c^

^a^Significantly different from men, *P* < 0.05.

^b^Significantly different from men, *P* < 0.01.

^c^Significantly different from men, *P* < 0.001.

All variables were expressed as the mean ± SD or the median and interquartile range (25th to 75th percentiles).

*Abbreviations*: *LDL* low-density lipoprotein, *Kt/V*_*urea*_ amount of dialysis delivered to each patient per treatment.

No significant sex differences were observed with respect to the age, dialysis vintage, or serum albumin levels of the study participants. However, the patient height, DW, BMI, and serum Cr levels were all shown to be significantly greater in men than in women. In addition, TMA and TMA standardized for DW (TMA/DW) were both significantly greater in men than in women, whereas TSFA and TSFA standardized for DW (TSFA/DW) were significantly greater in women than in men. No significant differences in the levels of CRP, sRAGE, and S100A12 were observed between men and women.

### Correlations between TMA/DW, TSFA/DW, sRAGE, and S100A12 levels and the clinical parameters

**Figure 1:**
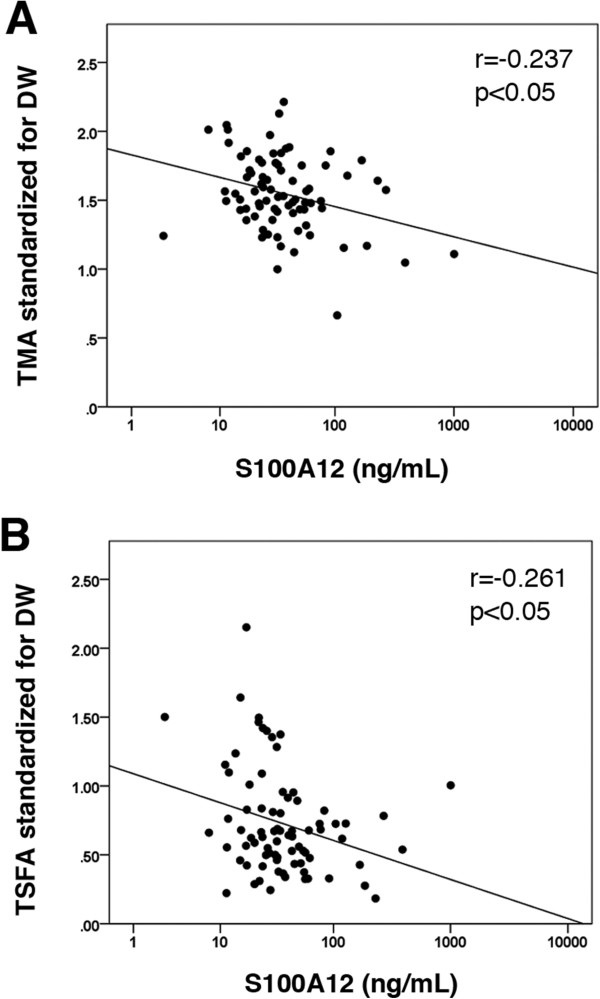
Associations between (A) TMA standardized for DW and plasma S100A12 levels and (B) TSFA standardized for DW and plasma S100A12 levels.

Significant positive correlations were observed between TMA/DW and the serum levels of albumin (*P* < 0.001) and creatinine (*P* < 0.001). In addition, TMA/DW was negatively correlated with age (*P* < 0.05) and Kt/V_urea_ (*P* < 0.05) (Table 2). TMA/DW was also negatively associated with CRP (*P* < 0.05) levels and with log-transformed S100A12 (*P* < 0.05, Figure 1A). Significant positive correlations were observed between TSFA/DW and the BMI (*P* < 0.05), total cholesterol (*P* < 0.01), LDL cholesterol (*P* < 0.05), serum creatinine (*P* < 0.001) and Kt/V_urea_ (*P* < 0.05) levels. However, TSFA/DW was negatively correlated with the CRP (*P* < 0.01) and log-transformed S100A12 (*P* < 0.05, Figure 1B) levels. Following log transformation, the sRAGE levels were shown to be negatively associated with BMI, but no significant associations were observed with TMA/DW and TSFA/DW, or the log-transformed S100A12 level. However, significant negative correlations were observed between the log-transformed S100A12 levels and HD vintage (*P* < 0.05), serum albumin (*P* < 0.01), TMA/DW (*P* < 0.05, Figure 1A), and TSFA/DW (*P* < 0.05, Figure 1B). In contrast, positive correlations were observed between log-transformed S100A12 levels and the levels of β_2_-microglobulin (*P* < 0.05) and CRP (*P* < 0.001).

**Table 2:** Correlations between TMA/DW, TSFA/DW, and Log sRAGE or Log S100A12 and Clinical Variables

	**TMA/DW**	**TSFA/DW**	**Log sRAGE**	**Log S100A12**
Age	−0.294^a^	−0.113	−0.032	0.138
HD vintage	−0.023	−0.066	0.115	−0.231^a^
BMI	0.033	0.266^a^	−0.232^a^	0.020
Total protein	−0.032	−0.013	0.088	0.033
Serum albumin	0.394^c^	0.206	0.117	−0.294^b^
Total cholesterol	0.177	0.341^b^	−0.092	−0.011
LDL chlesterol	0.080	0.270^a^	−0.120	0.023
Blood urea nitrogen	0.097	−0.154	−0.004	0.059
Serum creatinine	0.444^c^	0.042^c^	0.181	−0.111
Intact PTH	0.064	−0.022	−0.117	−0.147
β_2_-microglobulin	−0.151	0.074	0.209	0.282^a^
Kt/V_urea_	−0.203^a^	0.265^a^	0.151	−0.072
nPCR	0.040	−0.109	0.017	0.041
CRP	−0.269^a^	−0.343^b^	−0.211	0.478^c^
TMA/DW	-	−0.072	0.112	−0.237^a^
TSFA/DW	−0.072	-	0.033	−0.261^a^
Log sRAGE	0.112	0.033	-	−0.088
Log S100A12	−0.237^a^	−0.261^a^	−0.088	-

The data represent the correlation coefficients.

^a^*P* < 0.05, ^b^*P* < 0.01, ^c^*P* < 0.001.

*Abbreviations*: *TMA/DW* TMA standardized for DW, *TSFA/DW* TSFA standardized for DW, *LDL* low-density lipoprotein, *Kt/V*_*urea*_ amount of dialysis delivered to each patient per treatment.

### Determinants of potential biomarkers of muscle and/or fat mass

Multiple regression analyses revealed that log-transformed S100A12 was independently associated with TMA/DW and TSFA/DW when log-transformed S100A12, age, sex (model 1), HD vintage, BMI (model 2), total cholesterol, and CRP (model 3) were included as independent variables (Table 3).

**Table 3:** Multiple regression analysis with TMA standardized for DW or TSFA standardized for DW as the dependent variables and S100A12, Age, Sex, HD vintage, BMI, total cholesterol, and CRP as the independent variables

	**Model 1**		**Model 2**		**Model 2**	
**Variable**	**β**	***P***	**β**	***P***	**β**	***P***
**Dependent variable: TMA standardized for DW**						
Log S100A12	−0.305	0.004	−0.327	0.003	−0.312	0.004
Age	−0.242	0.019	−0.252	0.015	−0.215	0.034
Sex	0.369	<0.001	0.386	<0.001	0.4572	<0.001
HD vintage	-	-	−0.094	0.383	−0.094	0.371
BMI	-	-	−0.099	0.361	−0.122	0.247
Total cholesterol	-	-	-	-	0.227	0.031
CRP	-	-	-	-	−0.144	0.159
**Dependent variable: TSFA standardized for DW**						
Log S100A12	−0.191	0.026	−0.167	0.025	−0.181	0.014
Age	0.028	0.737	0.052	0.471	0.074	0.295
Sex	−0.659	<0.001	−0.744	<0.001	−0.695	<0.001
HD vintage	-	-	0.035	0.639	0.039	0.601
BMI	-	-	0.397	<0.001	0.378	<0.001
Total cholesterol	-	-	-	-	0.152	0.041
CRP	-	-	-	-	−0.042	0.563

Furthermore, when S100A12 levels were divided into three tertiles [S100A12 < 24.0 (*n* = 26), 24.0 ≤ S100A12 < 44.0 (*n* = 27), and S100A12 ≥ 44.0 ng/mL (*n* = 26)], then TMA/DW and TSFA/DW were significantly decreased (Table 4).

**Table 4:** Associations between TMA/DW, TSFA/DW and S100A12

	**S100A12 (ng/mL)**			
	**Tertile 1 (<24.0)**	**Tertile 2 (24.0 to 44.0)**	**Tertile 3 (≥44.0)**	
TMA standardized for DW	1.63 ± 0.23	1.60 ± 0.29	1.42 ± 0.27	*P* < 0.05
TSFA standardized for DW	0.88 ± 0.48	0.72 ± 0.36	0.58 ± 0.22	*P* < 0.05

The numbers in parentheses delineate the range for each S100A12 group.

Data of TMA and TSFA standardized for DW (TMA/DW and TSFA/DW, respectively) are shown as the mean ± SD.

## Discussion

The primary finding of this study is that high plasma levels of S100A12 are independently associated with low mid-thigh muscle mass and low subcutaneous fat mass in HD patients, even after adjusting for potential confounding variables. To our knowledge, this study is the first to determine the role that the RAGE system plays in the nutritional status of patients with advanced CKD.

Advanced CKD patients often suffer from nutritional problems that are associated with increased morbidity and mortality [27]. PEW is a term that has been proposed to describe the state of decreased body stores of protein and energy (i.e., muscle and fat mass) that occurs in CKD. In fact, HD patients have been reported to exhibit lower BMIs than age- and sex-matched control subjects from the general population [28]. Based on this observation, several studies have shown that increased BMI contributes to survival advantages in dialysis patients [8,9]. Because increased BMI has been associated with an increased risk of cardiovascular disease and all-cause mortality in the general population [7], this opposite relationship that is observed in dialysis patients is known as “risk factor paradox” or “reverse epidemiology” [29,30].

In dialysis patients, increased serum Cr levels have been associated with improved survival, whereas lower serum Cr levels have been associated with increased mortality [31-33]. This finding suggests that low serum levels of Cr as a proxy for low muscle mass could be associated with adverse outcomes [31]. Carrero et al. [34] also reported that muscle wasting measured by subjective global assessment (SGA) is associated with the increased mortality. These observations suggest that muscle mass is an important predictor of mortality in HD patients. However, increased fat mass has been associated with a lower risk of mortality and a reduced risk of hospitalization in HD patients [35]. Moreover, Kakiya et al. [36] showed that a decrease in body fat is associated with an increased risk of death in these patients. Currently, it remains to be determined whether the survival advantage associated with higher BMI in dialysis patients is caused by increases in muscle mass, fat mass, or both. One reason that this question remains unanswered is because BMI cannot differentiate between weight changes caused by muscle mass alterations and weight changes resulting from fat mass or water weight [8]. Previously, Beddhu et al. [37] attempted to investigate this issue using 24-hour urinary creatinine excretion as a surrogate for muscle mass. Based on their analysis, Beddhu and colleagues hypothesized that muscle mass might be a more important contributor to this survival advantage than fat mass. However, additional studies that directly measure muscle mass and fat mass are required to clarify this issue.

AGEs are generated as a result of chronic hyperglycemia and enhanced oxidative stress [38,39]. AGEs were initially thought to be the primary active ligands for their receptors, such as RAGE, but several new ligands, including the high-mobility group box proteins, S100 proteins, and amyloid fibrils, have been recently identified [40]. The binding of these ligands to RAGE induces oxidative stress, inflammation, and extracellular matrix accumulation [39,41]. Because the plasma AGE level changes that are observed in CKD patients are relatively modest [42], ligands other than AGEs for RAGE may be more important in developing RAGE-mediated complications in CKD. For example, S100A12, which is also known as EN-RAGE, has been identified as an interesting pro-inflammatory ligand for RAGE that triggers the RAGE pathway. S100A12 activates key inflammatory signals, such as nuclear factor-κB (NF-κB), and it stimulates the production of pro-inflammatory cytokines, such as IL-1β and TNF-α [18,20]. In contrast, sRAGE, which acts as a decoy receptor for RAGE ligands, suppresses RAGE-mediated inflammatory responses [17]. Recently, several studies have focused on the pivotal role of RAGE signaling in patients with CKD [15,21-23]. For example, sRAGE is negatively associated with systemic inflammation and with carotid intima-media thickness in PD patients [21]. In addition, S100A12 has been shown to predict the cardiovascular and all-cause mortality in HD patients [22,23]. In the present study, S100A12 was shown to be significantly and negatively associated with muscle mass and fat mass by both univariate and multivariate analysis, whereas no such relationships were observed for sRAGE. Thus, our study reveals the potential value of S100A12 as a predictor of nutritional status and provides clinical evidence regarding its possible role in the development of PEW in HD patients. In contrast, our data indicate that sRAGE levels are likely of limited clinical value in identifying PEW patients, although this possibility should be tested in a specifically designed clinical trial.

Another key finding from the present study is that the negative associations of S100A12 with muscle mass and fat mass both still remain significant even after adjusting for systemic inflammation (Table 3). As reported previously, systemic inflammation can cause a decrease in muscle mass because pro-inflammatory cytokines can contribute to anorexia, inhibit protein synthesis and promote catabolism [30]. Adipose tissue is a well-known source of pro-inflammatory cytokines, and obese CKD patients have actually been shown to exhibit higher levels of inflammation [43,44]. These findings highlight the difficulty of using inflammation as the sole explanation for the observed reductions of muscle and fat mass. Therefore, to clarify the influence of S100A12 on PEW in dialysis patients, we tried to adjust for the levels of systemic inflammation. Our analysis revealed that S100A12 is a direct predictor of muscle and fat mass independent of systemic inflammation. Previously, Hofmann et al. [45] demonstrated that S100A12 reduces cellular proliferation and increases H_2_O_2_ production via the NADPH oxidase system. Together, our results suggest that high levels of plasma S100A12 are associated with PEW via non-inflammatory mechanisms, such as oxidative stress. Further basic studies are warranted to clarify the precise role of this interesting ligand in determining nutritional status.

Our study has several limitations. First, due to the cross-sectional study design, a longitudinal causal relationship cannot be established between the changes in plasma S100A12 levels and alterations in muscle and/or fat mass. Second, because of the relatively small number of patients in our cohort, the generalizability of our conclusions remains unclear, and our data should be confirmed by larger studies.

## Conclusions

S100A12 is significantly and negatively associated with both muscle mass and fat mass in HD patients. Our findings suggest that plasma S100A12 levels could play an important role in determining the nutritional status of HD patients. Future longitudinal observations and interventional studies are warranted to establish whether this link is causal in nature.

## Abbreviations

AGE: Advanced glycation end product; BMI: Body mass index; CKD: Chronic kidney disease; Cr: Creatinine; CRP: C-reactive protein; CVD: Cardiovascular disease; DW: Dry weight; EN-RAGE: Extracellular newly identified RAGE-binding protein; HD: Hemodialysis; HMGB1: High-mobility group box 1; IL: Interleukin; Kt/V_urea_: Amount of dialysis delivered to each patient per treatment; LDL: Low-density lipoprotein; NF-κB: Nuclear factor-κB; PD: Peritoneal dialysis; PEW: Protein-energy wasting; RAGE: Receptor for advanced glycation end products; SGA: Subjective global assessment; sRAGE: Soluble RAGE; TMA: Thigh muscle area; TMA/DW: TMA standardized for DW; TNF: Tumor necrosis factor; TSFA: Thigh subcutaneous fat area; TSFA/DW: TSFA standardized for DW.

## Competing interests

The authors declare that they have no competing interests.

## Authors’ contributions

HF, HY, HK and RF designed the study. SI and NKK were involved in the acquisition of data. HF performed the statistical analysis and drafted the manuscript. All authors read and approved the final manuscript.
